# Continent-Wide Analysis of COVID 19: Total Cases, Deaths, Tests, Socio-Economic, and Morbidity Factors Associated to the Mortality Rate, and Forecasting Analysis in 2020–2021

**DOI:** 10.3390/ijerph18105350

**Published:** 2021-05-18

**Authors:** Muhammad Nauman Zahid, Simone Perna

**Affiliations:** Department of Biology, College of Science, University of Bahrain, Sakhir 32038, Bahrain; nzahid@uob.edu.bh

**Keywords:** COVID-19, continents, epidemiology, infection, deaths, risk factors

## Abstract

Background: The severe acute respiratory syndrome coronavirus 2 (SARS-CoV-2) was first reported in China in December 2019 and has become a pandemic that resulted in more than one million deaths and infected over 35 million people worldwide. In this study, a continent-wide analysis of COVID-19 cases from 31st December 2019 to 14th June 2020 was performed along with socio-economic factors associated with mortality rates as well as a predicted future scenario of COVID-19 cases until the end of 2020. Methods: Epidemiological and statistical tools such as linear regression, Pearson’s correlation analysis, and the Auto Regressive Integrated Moving Average (ARIMA) model were used in this study. Results: This study shows that the highest number of cases per million population was recorded in Europe, while the trend of new cases is lowest in Africa. The mortality rates in different continents were as follows: North America 4.57%, Europe 3.74%, South America 3.87%, Africa 3.49%, Oceania and Asia less than 2%. Linear regression analysis showed that hospital beds, GDP, diabetes, and higher average age were the significant risk factors for mortality in different continents. The forecasting analysis since the first case of COVID-19 until 1st January 2021 showed that the worst scenario at the end of 2020 predicts a range from 0 to 300,000 daily new cases and a range from 0 to 16,000 daily new deaths. Conclusion: Epidemiological and clinical features of COVID-19 should be better defined, since they can play an import role in future strategies to control this pandemic.

## 1. Introduction

Coronavirus disease 2019 (COVID-19) is an ongoing pandemic that resulted in global health, economic, and social crises [1]. It has created the worst health challenges since the Spanish flu in 1918 and the largest worldwide recession since the Great Depression [2]. This pandemic has led to the abandonment of all religious, political, sporting, cultural, and educational activities worldwide [3]. According to a report, 98.6% of students are affected globally due to the closure of schools, colleges, and universities [4]. According to the World Health Organization (WHO), this disease has already killed 1,042,344 people and affected 35,437,479 people worldwide by 29th September 2020 [5].

The World Health Organization (WHO) was informed on 31 December 2019 by China about many cases of pneumonia in Wuhan, China [6]. Initial cases of such pneumonia patients were reported on 8th December 2019 in Wuhan, China, and most of them were linked to the local Huanan South China seafood market of wild animals [7]. The cause of that pneumonia was identified on 7th January 2020 as a novel coronavirus (2019-nCoV), which was renamed as severe acute respiratory syndrome coronavirus 2 (SARS-CoV-2) and the disease was termed as coronavirus disease 2019 (COVID-19) by the WHO [5].

The sudden intrusion of COVID-19 in our life has shifted the focus of the world community, especially scientists, toward this virus. Despite rigorous efforts in microbiology, medicine, and pathology, many questions are still unanswered. With the help of a precise prediction of the further course of development, important countermeasures can be taken in risk management and communication [8]. In this study, epidemiological and statistical tools were being used to elaborate the current situation of COVID-19 patients and death rates in the different continents as well as forecasting analysis of COVID-19 cases enabling further understanding of the gravity of this pandemic. This work contributes to the academic world in two ways. First, a novel analysis of the new cases and deaths rate in different continents has been developed. Second, a forecasting analysis is applied to predict the new cases and the trend from 14th June 2020 to 1st January 2021.

## 2. Materials and Methods

The data were obtained from the European Centre for Disease Prevention and Control https://data.europa.eu/euodp/en/data/dataset/covid-19-coronavirus-data (accessed on 14 October 2020) and https://www.who.int/healthinfo/statistics/data/en/ (accessed on 14 October 2020). The time-series analysis in this study was based on the daily number of laboratory-confirmed cases reported from 31st December 2019 to the 14th of June 2020. The selected outcomes were the number of new and cumulative daily cases, deaths, rate of deaths, and number of population continent-wide. This study collected the following outcomes:

Total number of cases and deaths worldwide

- Diabetes Prevalence
- Stringency Index
- Median Age
- Handwashing Facilities
- GDP Per Capita
- Female Smokers
- Extreme Poverty
- Male Smokers
- Cardiovascular Diseases Death Rate
- Hospital Beds

### Statistical Analysis 

The statistical analysis and reporting of this study were conducted in accordance with the consolidated standards of reporting epidemiological studies guidelines [9]. Descriptive statistics reporting the daily number of new cases, daily deaths, and daily tests were performed and displayed in figures with line charts. Confounding variables were test “a priori” with Spearman correlation analysis. The primary risk factors associated with mortality rate in percentage were calculated with meta-regression stepwise analysis after testing for the linear and additivity of predictive relationships for independence (lack of correlation) of errors, testing for homoscedasticity (constant variance) of errors, and testing for normality of the error distribution. For the baseline variables, summary statistics employed frequencies and proportions for categorical data as well as mean for continuous variables represented in a graph. In this study, the Auto Regressive Integrated Moving Average (ARIMA) model, an advanced time series forecasting technique, was employed [10]. The data from all the countries were analyzed in the following manner:

(a) Inspection for stationarity using sequence charts and correlograms
(b) Differencing to transform non-stationary data to stationary
(c) Creation of ARIMA models based on the autocorrelation function (ACF) and partial autocorrelation functions (PACF)
(d) Determination of ARIMA (p,d,q) model fit
(e) Forecasting the time series for next few months i.e., until 31st December 2020

For the data analysis, an IBM SPSS Version 25.0 software (IBM SPSS statistics for windows version 20, Armonk, NY, USA) was used [11].

Statement for Ethical Approval: As this study is based on database and it did not involve human or animal experiments, therefore, ethical approval was not required for this study.

## 3. Results

**Figure 1:**
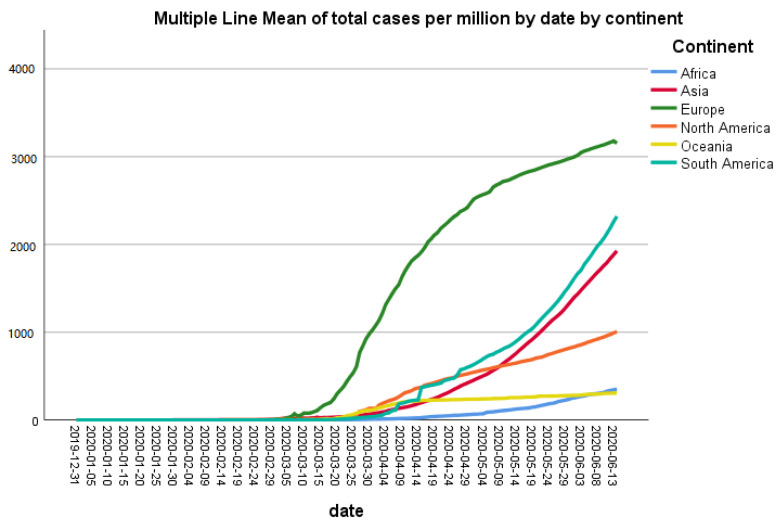
Trend of cumulative new cases standardized for population (per million) by continent.

From 31st December 2019 to 14th June 2020, the outbreak of coronavirus disease 2019 (COVID-19) caused 7,984,067 confirmed cases and 435,181 deaths in the world. After standardizing the data of COVID-19 positive cases per million population, the highest number of cases were recorded in Europe, while the lowest one was recorded in Oceania (Figure 1). The cases in South America started to rise in the beginning of May 2020 and as shown in Figure 1, it is still rising steeply. North America also showed a trend of escalation in the number of cases in the first week of May 2020. In other continents, cases are gradually increasing as well but at a slower rate than neighboring countries (Figure 1).

**Figure 2:**
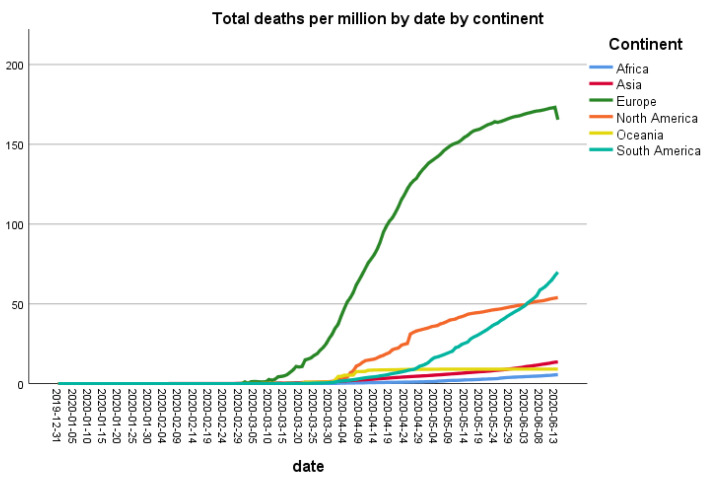
Trend of cumulative new deaths standardized for population (per million) by continent.

After standardizing the data of deaths per million population, the highest number of deaths were recorded in Europe followed by North America. The number of deaths in other continents is also increasing gradually from mid-May 2020, especially in South America (Figure 2).

**Figure 3:**
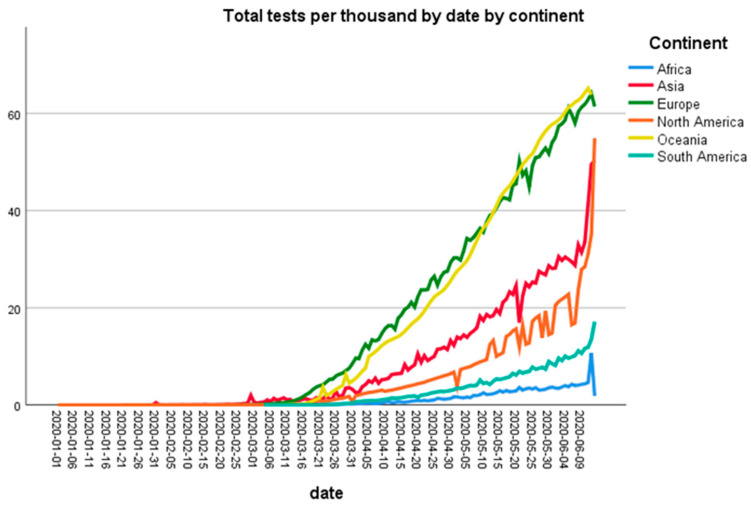
Number of cumulative coronavirus disease 2019 (COVID-19) tests (per thousand) by continent.

Figure 3 shows the number of tests performed by continents per thousand population. Oceania and European countries performed the highest number of tests per thousand population followed by Asia and North America.

Table 1 shows the risk factors associated to high mortality rate in African countries. Male smokers and high CVD death rate were the main risk factors associated to the mortality rate.

As shown in Table 2, the main risk factors associated to the mortality are the median age of the population, the smoke consumption (opposite for gender), handwashing facilities, population density and stringency index.

**Figure 4:**
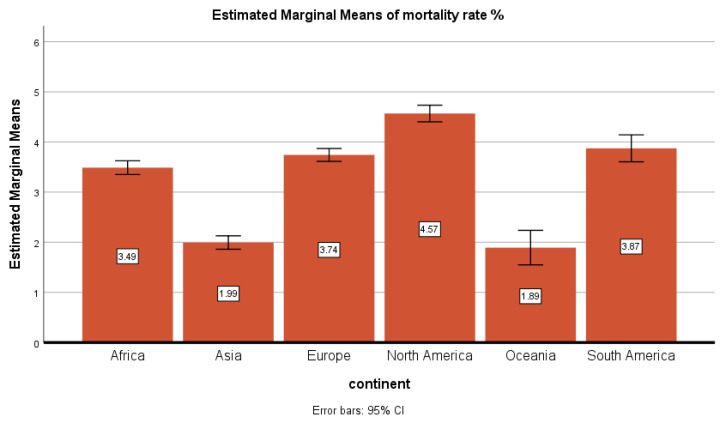
Mortality rate in percentage by continent.

Figure 4 showed the mortality rate percentage (cumulative cases/cumulative deaths) by continents. The mortality rate by continents was as follows: North America 4.57%, Europe 3.74%, South America 3.87%, Africa 3.49%, and Oceania and Asia, lower than 2%.

**Figure 5:**
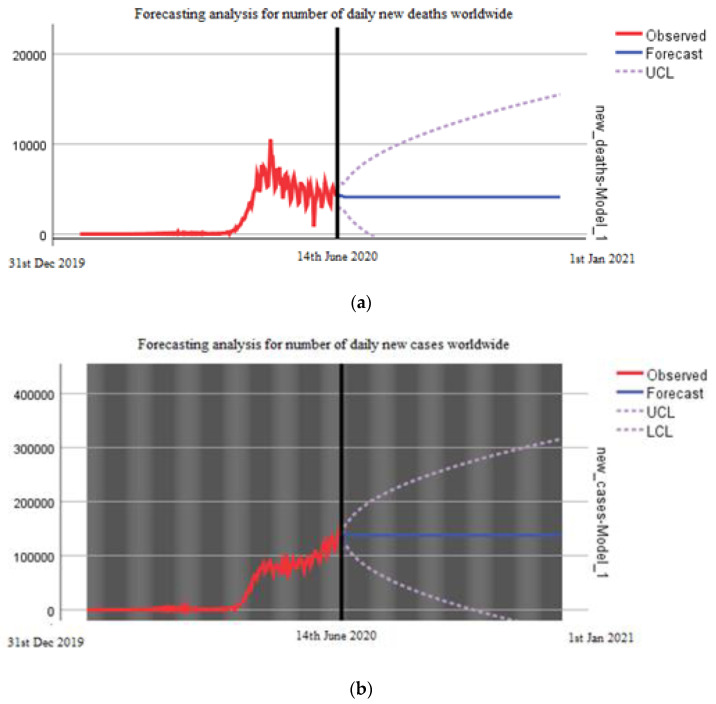
(**a**) Forecasting analysis for number of new daily deaths worldwide until 1st January 2021. (**b**) Forecasting analysis for number of new daily cases worldwide until 1st January 2021.

Figure 5a,b describes the forecasting analysis since the first case of COVID-19 wordwide. The data is predicted from 14th June 2020 to 1st January 2021. The worst scenario for the end of 2020 with the forecasting analysis predicts a range from 0 to 300,000 daily cases and a range from 0 to 16,000 daily deaths (Figure 5); since the data pattern did not demonstrate stationarity with first-order differencing, second-order differencing was done to achieve stationarity.

## 4. Discussion

By using epidemiological and statistical tools, we described the current situation of COVID-19 positive cases and deaths in different continents as well as the main risk factors associated with mortality rate and worldwide forecasting analysis that predicted the scenario of the COVID-19 pandemic until the end of 2020. This study showed that mortality rate is associated with different factors in different countries including smoking, cardiovascular diseases (CVD), diabetes, hand washing, and number of beds in hospitals per thousand population. Secondly, this epidemiological study sheds light that after standardizing the data of cases per million population, the highest number of cases were recorded in Europe, while the trend of new cases is lowest in Africa.

These data indicate that COVID-19 has severely affected Europe, as there is a huge gap in the number of COVID-19 positive cases per million population in Europe and the rest of the continent, but it seems that the steepness of the curve has decreased now. In contrast to Europe, the number of positive cases is increasing in Asia and South America. Although the number of cases in China has reduced, other countries of Asia such India and Pakistan have been under severe threat of COVID-19 from the beginning of June 2020. As these countries are highly populated, Asia could become the next hotspot of COVID-19. Interestingly, Oceania and Africa showed very low number of cases per million population. One reason for the low number of cases in Africa seems to be a smaller number of testing in this continent, whereas Oceania conducted the highest number of tests per thousand inhabitants. It is important to find out the reasons for this low number of tests in most countries. The standard diagnostic test for COVID-19 diagnosis is RNA-RT-PCR assay, which has been recommended by the WHO on nasopharyngeal swabs [12]. This test is very expensive and governments of many poor countries, such as in Africa, cannot afford to provide it for free for all citizens. The serological tests are relatively cheap and quick, but broad use of these tests for diagnostic purpose is still controversial. To test the efficacy of these serological tests, Lahner et al. have performed IgM/IgG antibody-based serology tests in 1084 samples from heath workers. They found a 98.99% specificity of IgM serology, while IgM showed 99.1% specificity. This study described that the performance of IgG serology tests was better two weeks after the infection of COVID-19 detected by RNA-RT-PCR [12]

The reason for these differences in cases among different continents will be very crucial to understand, as that might help control the spread of this pandemic. The COVID-19 pandemic has affected almost all countries of the world, but surprisingly, the intensity of disease is not the same everywhere. According to the WHO data, the number of cases per million population in the USA, Italy, and the UK are 5268, 3847, and 3996, respectively [5]. Among South American countries, COVID-19 is spreading quickly in Brazil, as the number of cases per million population there is 2345. Interestingly, China, from where this pandemic started, is in recovery phase, so its number of cases per million population is 57 [5].

Astonishingly, there is a huge difference in the number of deaths among different countries as well as different continents [5]. The data of deaths per million population showed that the highest number of deaths were recorded in Europe followed by North America and South America. For example, the number of deaths per million population in Italy and the UK are 551 and 562, respectively, while in the USA, it is 310 [5]. Similarly, Brazil also showed the high number of deaths, i.e., 133 while in China, there are 3.2 deaths per million population [5]. Surprisingly, Africa, where the health system is weak compared to Europe and America, observed a very low number of deaths during the above-mentioned study period. It will be important to identify the reason(s) for this low number of deaths in Africa, as it may be the critical factor to control the disastrous effects of this pandemic. The exact reason for this difference in death rates is also still unknown, as this virus is only 5 months old, and many aspects of the viral genome and pathogenesis are still under study. However, mutations in the viral genome could be one of the reasons of the variability in the severity of this virus. Dr. Rodney P. Jones described different factors involving high mortality due to COVID-19 in the USA and the UK. He linked high mortality with population density, as it results in household crowding and poor hygiene. Another important factor is the capacity pressure on hospitals due to which patients could not find beds, and it was hard to take care of such patients. Dr. Rodney further explained that the cognitive dissonance and high granularity of COVID-19 also increased mortality [13]. Interestingly, it has been reported that COVID-19 spread and air pollution have a positive correlation. As it is known that COVID-19 can be transmitted through air; therefore, atmospheric particulate matter (PM) can transport the SARS-CoV-2 greater distances than those believed for only close contacts. This PM is also responsible for inflammation in lung cells, so people in more polluted areas have more probability of getting severe symptoms of COVID-19, leading to more mortality [14].

We have also described the mortality rate in different continents. It was observed that South America had the highest mortality rate followed by Europe and North America. The mortality rate in some countries is very high such as in Italy and the UK, where the mortality rate is 14% and 14.6%, respectively. The USA has a 6% mortality rate and Brazil shows a 6.5% mortality rate, while China has only a 3.2% mortality rate [5]. This is also surprising because the health system in most of the countries of these continents is far better than the health system of Africa and Asia, but still, the mortality rate in these continents, especially in Asia, is very low. Interestingly, it was speculated initially that hot weather may be a reason for less mortality in Asia and Africa, but the current situation in Asia has also nullified this theory, because the cases and death rates are increasing here in June, which is one of the hottest months in this region [15,16]. Moreover, Oceania showed the lowest mortality rate, and even New Zealand has claimed to be free of COVID-19 cases, although the weather is cold there in June. It shows that weather does not play a vital role in the COVID-19 pandemic. All these findings are speculations until we get more information about the viral genome throughout the world.

Regarding the risk factors associated to mortality, the strongest one was the number of hospital beds, which showed a high relevance and impact on mortality. The number of beds and the healthcare facilities had an important impact during this emergency, specifically in Europe, where many elderly people were admitted to the unit of intensive care. Surprisingly, the number of handwash facilities and the average age does not affect the rate mortality in many continents. It is important to discuss the impact of COVID-19 on rural areas, as those areas have badly suffered from previous pandemics such as H1N1. The lack of facilities such as advanced intensive care units (ICUs) and ventilators have raised the mortality rate in rural areas. Rural areas also have the issue of professional resources as they have a low number of healthcare workers, not enough big hospitals, a low number of physicians, and the older population is high in rural areas as compared to urban areas. All these factors have raised concern that COVID-19 can have a severe impact in rural areas of different countries [17].

Everyone around the world wants to know the end date of this pandemic, as it is required for future planning in all aspects of life. The evolution of virus is not totally haphazard, as we know this from previous pandemics. It follows a pattern from outbreak to the acceleration phase, plateau, and then decline phase, which is finally followed by the end of the pandemic. Using forecasting analysis, we have predicted the outcomes of the COVID-19 pandemic worldwide in both the best and worst scenarios. According to this model, if strict measures are continuously adapted, the trend will not surpass 100,000 daily new cases worldwide and no more than 5000 daily deaths. We predicted that in the worst scenario, there could be 300,000 cases per day at the end of this year while 16,000 deaths could be recorded. This tells us the seriousness of this pandemic, and all countries should implement strict measures to stop the spread of this disease. Although the situation in some countries is improving, the WHO warned about the second wave of the COVID-19 pandemic that could be more devastating [5]. Different groups have also performed forecast analysis on COVID-19, and the important aspect in all is that all such predictions depend on an accurate count of symptomatic and asymptomatic cases as well as the exact number of deaths caused by COVID-19. Moreover, these predictions also depend on the strict measurements taken by all countries, human behavior, and testing protocol; otherwise, with a change in the number of cases, there will be change in the predictions about the end of this pandemic [18,19,20,21]. For example, Jianxi Luo performed a data-driven prediction of next developments and end dates of COVID-19 in different countries. His analysis predicted the COVID-19 scenario in Brazil and USA, and it showed two different conditions i.e., a stable prediction in the USA, while in Brazil, the situation is highly volatile and needs more cautious actions [21]). In contrast, strict restrictions applied by the government of Singapore may bend the curve earlier than predicted [21]. Therefore, the forecasting analysis should be considered together with the situations in each country.

Several studies with different predictive models showed a similar prediction [22]. In particular, the US national ensemble forecast indicates an uncertain trend in new COVID-19 cases reported over the next four weeks (October–November 2020) and predicts that 160,000 to 360,000 new cases will likely be reported (https://www.cdc.gov/coronavirus/2019-ncov/cases-updates/forecasts-cases.html, accessed on 14 October 2020).

Finally, a recent study that took in account five of the models—IHME, YYG, Delphi, SIKJalpha and LANL—had less than 20% MAPE at six weeks. Despite the complexities of modeling human behavioral responses and government interventions related to COVID-19, predictions among these better-performing models were surprisingly accurate. Forecasts and alternative scenarios can be a useful input to decision-makers, although users should be aware of increasing errors with a greater amount of extrapolation time and corresponding steadily widening uncertainty intervals further in the future [23].

## 5. Conclusions

The situation of COVID-19 is changing every day in all continents. We have reported in this study that the number of cases as well as death rates is increasing gradually with time in most of the continents, while in some continents such as Asia, the number of COVID-19 positive cases rose up steeply in May 2020. Similarly, the death rates in these continents also jumped high in May 2020. The exact reasons behind the difference in the number of cases and casualties in different countries of the world is still unknown, since the factors that we investigated gave contradictory information. Further studies are required to understand COVID-19 for its characterization and development of a vaccine against this pandemic.

## Figures and Tables

**Table 1:** Socio-economic risk factors associated to mortality rate.

**Model ^a^ Africa**	**B**	**Std. Error**	**Beta**	**t**	***p* Value**	**CL 95%** **Lower Bound**	**CL 95%** **Upper Bound**
Stringency index	0.055	0.006	0.577	9.941	0.0001	0.044	0.066
Female smokers	−0.684	0.060	−0.289	−11.351	0.001	−0.802	−0.565
Hospital beds per thousand	2.188	0.295	0.435	7.426	0.001	1.610	2.765
Diabetes prevalence	−0.453	0.044	−0.429	−10.397	0.001	−0.538	−0.367
CVD * death rate	0.016	0.002	0.690	9.347	0.00001	0.013	0.020
Median age	−0.184	0.031	−0.581	−5.954	0.001	−0.245	−0.124
Male smokers	0.039	0.014	0.173	2.722	0.0001	0.011	0.067
**Model ^b^ Asia**	**B**	**Std. Error**	**Beta**	**t**	***p* Value**	**CL 95%** **Lower Bound**	**CL 95%** **Upper Bound**
CVD death rate	0.018	0.001	1.328	16.435	0.0001	0.015	0.020
Hospital beds per thousand	−1.116	0.062	−0.819	−17.941	0.0001	−1.239	−0.994
GDP per capita	0.000	0.000	0.324	5.768	0.0001	0.000	0.000
Diabetes prevalence	−0.612	0.070	−0.972	−8.748	0.0001	−0.750	−0.475
Female smokers	−0.262	0.042	−0.301	−6.178	0.0001	−0.345	−0.179
Extreme poverty	0.067	0.015	0.206	4.535	0.0001	0.038	0.097
**Model ^c^ Europe**	**B**	**Std. Error**	**Beta**	**t**	***p* Value**	**CL 95%** **Lower Bound**	**CL 95%** **Upper Bound**
Diabetes prevalence	0.399	0.014	0.898	28.043	0.0001	0.371	0.427
**Model ^d^ North America**	**B**	**Std. Error**	**Beta**	**t**	***p* Value**	**CL 95%** **Lower Bound**	**CL 95%** **Upper Bound**
Diabetes prevalence	1.804	0.081	3.423	22.330	0.0001	1.645	1.962
Handwashing facilities	−0.185	0.009	−2.742	−21.349	0.0001	−0.202	−0.168
Stringency index	0.085	0.005	1.371	15.429	0.0001	0.074	0.095
CVD death rate	−0.022	0.001	−1.100	−15.092	0.0001	−0.025	−0.019
GDP per capita	0.000	0.000	−0.387	−5.409	0.0001	0.000	0.000
**Model ^e^ South America**	**B**	**Std. Error**	**Beta**	**t**	***p* Value**	**CL 95%** **Lower Bound**	**CL 95%** **Upper Bound**
Stringency index	0.057	0.005	1.152	11.344	0.001	0.047	0.067
Female smokers	−0.840	0.080	−0.812	−10.561	0.001	−0.997	−0.684
Median age	0.071	0.017	0.483	4.087	0.0001	0.037	0.105

^a^ continent = Africa, * CVD = cardiovascular disease; ^b^ continent = Asia. Table 1 shows that in Asian countries, high mortality rate is associated with hospital beds per thousand population, diabetes prevalence, and female smokers; ^c^ continent = Europe. In European countries, the diabetes prevalence appears to be the only risk factor associated with high mortality rate (Ɓ = 0.014; *p* > 0.0001); ^d^ continent = North America. Table 1 explains that in North America, handwashing facilities and CVD death rates are linked with high mortality rate; ^e^ continent = South America. In South American countries, the age of the population appears to be the main risk factor associated to the high mortality rate (Ɓ = 0.014; *p* > 0.0001).

**Table 2:** Overall risk factors associated to mortality rate worldwide.

Predictor	Estimate	SE	t	*p*	Stand. Estimate	Lower	Upper
stringency_index	0.09904	0.008	12.38499	0.0001	0.17574	0.1479	0.2036
population_density	−0.00507	0.00158	−3.20247	0.0001	−0.05527	−0.0891	−0.0214
gdp_per_capita	−6.00 × 10^−5^	7.4	−0.811	0.4174089	−0.01928	−0.0659	0.0273
median_age	0.30829	0.08542	3.60924	0.0001	0.11611	0.053	0.1792
extreme_poverty	−0.02046	0.02429	−0.8424	0.3996054	−0.02352	−0.0783	0.0312
cvd_death_rate	−6.05 × 10^−4^	0.00316	−0.19119	0.8483846	−0.00366	−0.0412	0.0339
diabetes_prevalence	0.00106	0.10746	0.00989	0.9921134	2.07 × 10^−4^	−0.0408	0.0413
female_smokers	0.29986	0.05808	5.16296	0.0001	0.08176	0.0507	0.1128
male_smokers	−0.16995	0.01838	−9.24648	0.0001	−0.16645	−0.2017	−0.1312
handwashing_	0.07807	0.02128	3.66916	0.0001	0.13767	0.0641	0.2112
